# Supplementary material for: Vitamin D Modified DSS-Induced Colitis in Mice via STING Signaling Pathway
Source: Biology (Basel). 2025 Jun 18;14(6):715. doi: 10.3390/biology14060715 (PMC12190092; doi:10.3390/biology14060715)
Supplement: Supplementary file 1 [file biology-14-00715-s001.zip › Supplementary Materials S1: 16s-report of gut microbiota/Groups/diff_analysis/UnivarTestGBM/figure/boxplot/top10.pdf]

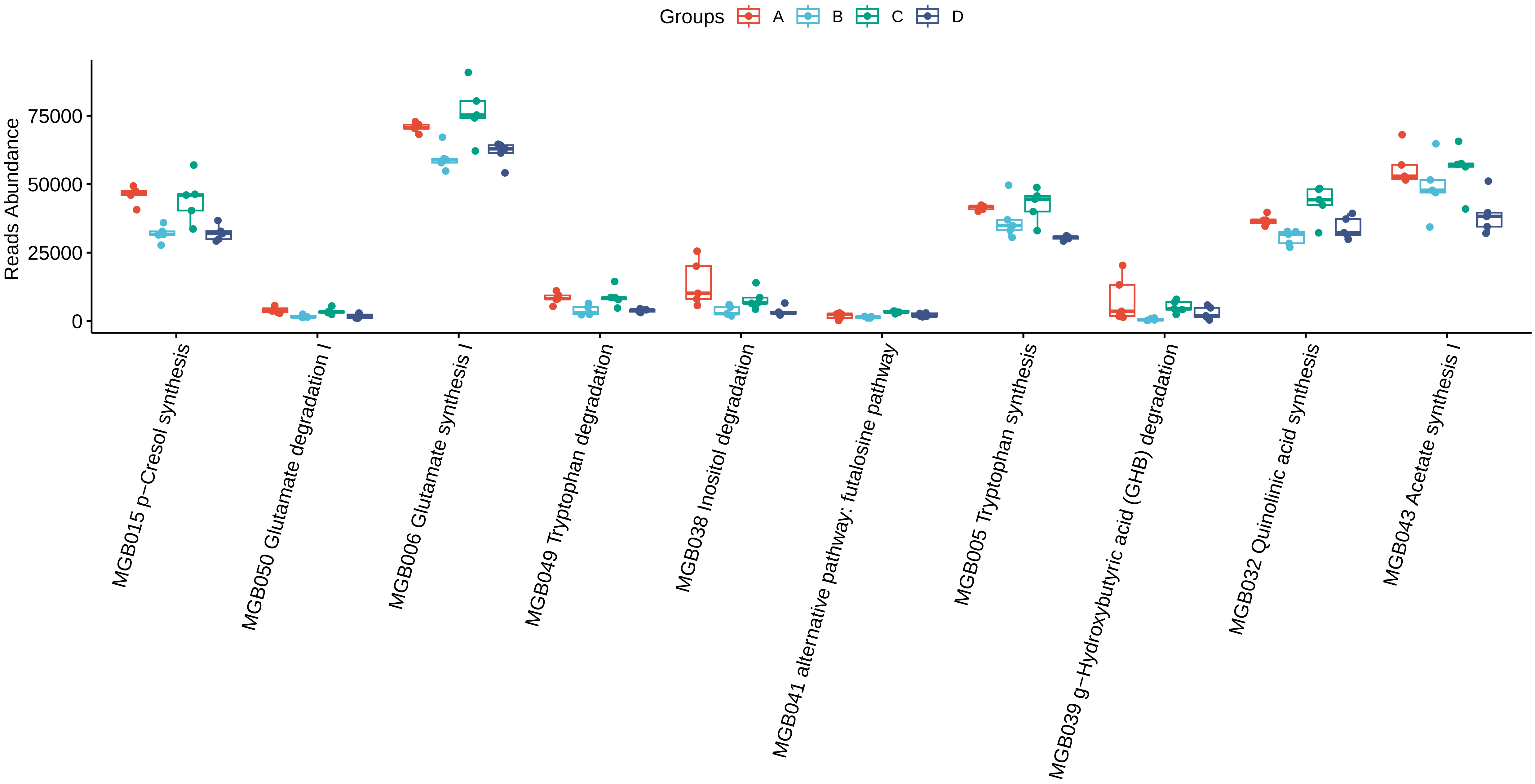

Groups
A
B
C
D
Reads Abundance
75000
50000
25000
0
MGB015 p−Cresol synthesis
MGB050 Glutamate degradation I
MGB006 Glutamate synthesis I
MGB049 Tryptophan degradation
MGB038 Inositol degradation
MGB041 alternative pathway: futalosine pathway
MGB005 Tryptophan synthesis
MGB039 g−Hydroxybutyric acid (GHB) degradation
MGB032 Quinolinic acid synthesis
MGB043 Acetate synthesis I
